# Development and Evaluation of a Measure for Social Support Provided by Friends during Lifestyle Management Programs

**DOI:** 10.3390/healthcare10050901

**Published:** 2022-05-13

**Authors:** Laurie Abbott, Jennifer Lemacks, Tammy Greer

**Affiliations:** 1 College of Nursing, Florida State University, Tallahassee, FL 32306, USA; 2 College of Nursing and Health Professions, University of Southern Mississippi, Hattiesburg, MS 39406, USA; jennifer.lemacks@usm.edu; 3 School of Psychology, University of Southern Mississippi, Hattiesburg, MS 339406, USA; tammy.greer@usm.edu

**Keywords:** social support, lifestyle program, instrument, friends

## Abstract

Obesity is a public health crisis that contributes to chronic disease prevalence, morbidity, and mortality. Nutrition and physical activity are risk factors for many chronic diseases including cancer and cardiovascular disease, the leading causes of death in the United States. Lifestyle management programs to address obesity and potential sequelae such as chronic conditions have shown efficacy, with social support an important factor in interventions. Instruments that assess social support specifically provided by friends are lacking but could be important predictors of program success. The purpose of this study was to examine the reliability and validity of the 10-item Social Support to Eat Better and Move More instrument that was developed and designed to measure support from friends that influence dietary and physical activity behaviors during lifestyle management programs. Data were collected during a cross-sectional study using purposive sampling strategies among adult residents of two southern states. Statistical analysis was conducted to examine latent factors, internal consistency, and convergent and predictive validity. These preliminary results indicated that the Social Support to Eat Better and Move More instrument had excellent internal consistency for the overall measure (α = 0.96) as well as for informational support (α = 0.97), emotional support (α = 0.96), and encouragement (α = 0.97). The tool related well to another general social support measure as well as to diet, physical activity, and health-related variables, and it can be a useful measure in lifestyle management studies.

## 1. Introduction

In the United States, chronic diseases such as heart disease, cancer, and diabetes are the leading causes of mortality, morbidity, and high health care costs [1,2]. Approximately 60% of adults have at least one chronic disease, and 40% have been diagnosed with at least two [1,2]. The risk for developing a chronic disease is influenced by factors including obesity, diet, physical activity, and alcohol and tobacco use [2]. The percentage of adults considered obese has increased among both men (from 27.5% in 2000 to 43% in 2018) and women (from 33.4% in 2000 to 41.9% in 2018), and this public health problem is especially prevalent among people living in the southern states [3,4]. The links among obesity, poor nutrition, and being sedentary have been well established in the literature, and all three are chronic disease risk factors, individually and collectively [3,4].

Lifestyle management interventions that promote healthy lifestyle choices can facilitate improvements in dietary and physical activity behaviors that can potentially reduce the prevalence of obesity and chronic disease in high-risk areas [3]. However, modifying dietary and physical activity behaviors is multi-faceted and involves several levels of influence. To help explain the complexities involved with changing health behaviors, the socio-ecological model can be adapted for health promotion and disease risk prevention [5]. In this context, lifestyle behaviors are influenced by the interactions of multiple interdependent factors at various levels including intrapersonal/individual, interpersonal, community, institutional/organizational, and public policy [5]. At the individual and intrapersonal levels, social support can be considered an important aspect of health behavior change and can facilitate improvements in chronic disease risk factors, including nutrition and physical activity outcomes [6,7,8]. These concepts of social support serving as determinants of intentions to engage in health behavior change are supported by other health behavior change theories including the social cognitive theory and the theory of planned behavior [9,10,11].

People with higher levels of social support typically have inversely lower rates of chronic diseases such as diabetes, hypertension, and stroke [12]. Social support is defined as a function of social networks or relationships that promotes positive feelings of being appreciated by others [13,14]. Meaningful connections and relationships with others, such as family and friends, involved with social support induce perceptions of belongingness within a group [12]. In the context of health education and behavior, social support is categorized into four main types including emotional, instrumental, informational and appraisal support [15]. For health behavior change, important aspects of social support are emotional support that involves caring and trust by those within social relationships and informational support as a means of providing information to others that can facilitate decision making [15]. Social support in the form of encouragment from friends can faciliitate improvements in dietary outcomes [6].

Having social support specifically from friends has been associated with improvements in both nutrition and physical activity outcomes [16]. While there is strong evidence to suggest the importance of social support from family members, there is a dearth of measures particularly focused on the social support provided by people other than family, such as friends, that can facilitate nutrition and physical activity behavior changes among adults during lifestyle management programs. We postulate the three types of non-family support that are necessary components for lifestyle management programs are informational support, emotional support, and encouragement. Thus, the purpose of this study was to examine the reliability and validity of a tool to measure support from friends that influence dietary and physical activity behaviors during the implementation of lifestyle management programs. In addition to the socio-ecological model, this study was guided by the social networks and social support framework, which explains that social support positively influences health when the basic human needs for relationships with others are met by enhancing the ability to cope and reducing stress-related health issues [14].

## 2. Materials and Methods

This study is an analysis of a subset of data from a cross-sectional parent study conducted in June 2020 using purposive sampling strategies among residents of two southern states. The purpose of the parent study was to describe experiences and attitudes regarding the COVID-19 pandemic and determine predictors of adherence to stay-at-home orders [17].

### 2.1. Study Setting and Participants

Participants were included if they were (a) adults 18 years of age and older, and (b) residents of Mississippi or Louisiana. Recruitment methods involved advertising via social media posts including Facebook and Instagram. The announcements were also added to professional and organizational social media pages such as the Mississippi INBRE Telenutrition Center and the Center for American Indian Research and Studies. Lastly, this study was also promoted through word of mouth by a university group, the Mississippi INBRE Outreach Scholars, and community partners. Eligible adults provided informed consent and completed the online survey for the parent study which consisted of measures for nutrition, physical activity, COVID-19, and preventable chronic disease. After completion of the survey, study participants were offered a 5 USD Walmart electronic gift card to thank them for their time and participation. All study procedures were reviewed and approved by the institutional review board at The University of Southern Mississippi.

### 2.2. Measures

The socio-demographic survey items were those typically collected in health-related research and included age, race/ethnicity, gender, education level, household income, marital status, and state of residence. The Social Support to Eat Better and Move More instrument, developed by the authors for the present study, included ten positively worded items designed to address three subdomains: informational support (3 items), emotional support (4 items), and encouragement (3 items). Responses were measured using a 7-point Likert-type scale with anchors ranging from strongly disagree to strongly agree. Informational support items included the stem, “When I need information to help me eat better or move more, I have a friend who…” followed by “… I can turn to for information,” “… will help me get the information I need,” or “…will give me ideas/things to try.” Emotional support items included the stem, “When I am trying to eat better or be more active, I have a friend who …” followed by “… cares about how I am doing with my goals,” “… I can connect with who is trying to do the same thing as me,” “… helps me problem solve,” or “encourages me to meet my goal(s).” Encouragement items included the stem, “When I meet my goals to eat better or be more active, I have a friend who …” followed by “… I can share my success with,” “… will celebrate my achievements with me,” or “… will be proud of me”.

As part of the survey development process, internal consistency, latent factors, and convergent and predictive validity were assessed. To assess validity, the three subdomains were related to a common and more general social and emotional support measure as well as to diet, physical activity, and physical health variables. Social and emotional support were measured using a five-item Likert response question from the Behavioral Risk Factor and Surveillance System that assessed how often social and emotional support were received on a scale of never to always [18]. Dietary variables, including total added sugars and fruit and vegetables intake, were collected using the Dietary Screener Questionnaire and calculated based on published algorithms [19]. Physical health was captured with the SF-12 that assessed perceived physical health using 5 items on a Likert-type scale with anchors ranging from poor to excellent [20]. Physical activity was assessed using a one-item question “In the past week, how many days have you done a total of 30 min or more of physical activity that raised your breathing rate?” with options ranging from 0 to 7 days [21].

### 2.3. Statistical Analysis

Descriptives were computed and analyses were conducted using IBM SPSS Statistics 27.0 software [22]. Principal component analysis was conducted to determine the latent structure of the measure. Cronbach’s alphas were computed to determine internal consistency for the domain and within subdomains of the new measure. Inter- and intra-item correlations were used to examine relationships across and within subdomains. Convergent and predictive validity were assessed by correlating the overall score and subdomain scores with general social and emotional support, total added sugar intake, fruit and vegetable intake, physical activity, physical health status, and self- and immediate family chronic disease status scores.

## 3. Results

Study participants (*n* = 368) ranged in age from 18 to 79 (M = 33.9, SD = 14) and were predominately female (75%). Almost two-thirds completed a 2 or 4 year degree (57.6%) and had household incomes (66%) at or above 40,000 USD. Approximately half of participants were married or cohabitating (49.4%). The socio-demographic characteristics of the sample are further described in Table 1.

Descriptives for items within proposed sub-domains of the Social Support to Eat Better and Move More measure are located in Table 2. Item averages ranged from 5.21 to 5.90, with values of 5 indicating a “somewhat agree” response. Over half of participants “agreed” or strongly agreed” with each of the positively worded social support statements.

Simple correlations among items and rotated loadings from a principal components analysis are included in Table 3. Correlations among informational support items ranged from r = 0.89 to r = 0.97, among emotional support items from r = 0.84 to r = 0.88, and among encouragement items from r = 0.91 to r = 0.94. Inter-item correlations among items in different subdomains ranged from r = 0.60 to r = 0.77, somewhat lower than interitem correlations within subdomains, indicating that subdomain items were more related to one another than to items outside their subdomains and providing some evidence that supports proposed subdomains. A principal components analysis on all 10 items of the Social Support to Eat Better and Move More measure with a varimax rotation, however, indicated only two factors with eigenvalues >1. The first eigenvalue explained 76.36% of the variance and the second vector explained only 10.21% of the variance. The three items designated as information support items loaded on the first factor and the three items designated as encouragement items loaded on the second factor. Items designated as emotional support had mixed loadings with near equal loadings on both factors. Cronbach’s α for the entire measure as well as for proposed subdomains was greater than 0.95 indicating high internal consistency among items. The rotated component matrix and Cronbach’s α values are in Table 3.

Convergent and predictive validity for Social Support to Eat Better and Move More measure and subdomains were correlated with added sugar intake, fruits and vegetable intake, physical activity, and perceived physical health. Only the encouragement support subdomain was not significantly associated with fruit and vegetable intake or physical activity. Results are shown in Table 4.

## 4. Discussion

The participants in this study included a larger percentage of women (75%) than men (25%). Further, the majority (83.1%) had attended some college or earned a college degree. The sample was more diverse regarding marital status, household income, and race/ethnicity. The literature suggests that social support from family members has an important role in heath behavior change interventions. However, the support provided by friends has been underrecognized and undermeasured in health research efforts and may differ from the type of support provided by family members. The Social Support to Eat Better and Move More instrument was developed to measure social support that is provided specifically by friends. The 10-item measure was found to be valid and internally consistent (α = 0.96). The evidence was mixed regarding the proposed subdomains of informational support, emotional support, and encouragement. Factor analysis results indicated that the items within each of the three subdomains were mostly related to other items within their category with emotional support having mixed loadings. Correlations were higher among items within compared to between sub domains. The overall measure and most subdomains were correlated with a more broadly defined emotional social support measures and self-report health behavior variables (diet, physical activity, etc.) as would be expected. Although more work is needed on the subdomains of the overall measure, findings for the measure clearly indicate the potential of Social Support to Eat Better and Move More to assess social support specific to friends.

The Social Support to Eat Better and Move More measure of social support is timely as health disparities research and interventions are increasingly addressing cultural and community-informed barriers and facilitators of healthy lifestyles. Our prior qualitative inquiries support the idea that participants of group-based lifestyle management programs gain support from friends, also called peers, who are participating in the program with them [23]. These findings were supported by a qualitative study among African American men that illustrated the importance of social connections in enhancing resources, sharing experiences and successes, and overcoming lifestyle change challenges [24]. Another study among African American participants showed that encouragement from friends was associated with health behavior improvements such as increases in fruit and vegetable intake [6]. Support groups containing friends may be critically important to achieving health behavior change, especially when the availability of family social support is minimal or limited. Another study reported that men were more likely to report engaging in positive health behaviors after receiving reinforcement from peers, such as congratulating them for achieving desired lifestyle changes and encouraging them to continue adopting healthy behaviors [25]. These findings highlight the special role that friends, or peers, have in facilitating health behavior changes among men and women participating in lifestyle management programs. The influences can be especially useful for interventions targeting minority men who have been underrepresented and difficult to engage in health disparity research, especially involving programs for dietary changes and weight loss [26,27]. Even male representation in survey data is limited as can be seen in our study by the larger number of females (*n* = 276) who agreed to participate compared to men (*n* = 92). Given the increasing rates of obesity linked with the high rates of chronic disease prevalence, morbidity, and mortality among both men and women [1,2,3,4], exploring strategies that facilitate greater intervention efficacy and vigorously measuring the influential social support factors associated with outcomes is imperative for moving the needle of health disparities among minorities in a positive direction.

Support from various types of social relationships should be differentiated to better identify the impact of different types of support on lifestyle management program outcomes and how they uniquely predict aspects of health behaviors and strategies for modifying them. For example, women living in underserved rural areas may have higher stress levels and lower levels of individual resilience when they lack social support [28]. Further, people with higher stress levels have greater chronic disease risk and are more likely to be obese, smoke, and have hypertension [29]. However, having social support may serve as a mediator that buffers the effects of stress [30]. These associations highlight the position that social factors can potentially change how adults perceive and respond to lifestyle interventions, whether positively or negatively, and the differing categories of support should be adequately identified, measured, and addressed in health and health disparity research. Most social support measures have combinations or subcategories of differing types of social support within the same measure, and it can be difficult to definitively pinpoint the relationships that contributed to intervention effects. As an example, a study showed that participants had improved physical activity and dietary outcomes, but the measure did not allow differentiation for whether the outcomes came from peers within the program or from other social relationships [16]. The availability of the Social Support to Eat Better and Move More instrument has the potential to fill that gap because it is a reliable tool to measure the specific impact that friends, or peers, may have on health program outcomes that can differ from familial support. Although family support can be influential for effecting health behavior changes, having support from friends may offer a different motivational source that should be explored and accurately measured to facilitate future intervention development and implementation.

This study had some limitations and implications for future research. Although screening methods were used to ensure the validity of the data, participants self-identified for this study and may not have been actual residents of the targeted areas. The results of the survey provided information about a specific region in the southern United States, and findings may differ in other geographic locations. However, because of the lack of valid surveys that measure support from other people besides family in health behavior research, the description of this survey, including its psychometric results, provides information for further health behavior change interventions. This study reported high values of alpha (>0.90), which may suggest redundancies and the need to shorten the length of the test [31]. Additionally, the principal components analysis indicated that only two factors with loadings that were mixed on the Encouragement subdomain. Therefore, future research could further define subdomains and develop and test more instruments intended to measure the social support provided by others including interventionists and research support staff during health behavior change programs. More work is needed to follow-up on our findings from focus group data that indicated that participants felt that support provided by friends was important and filled a void [23]. However, research efforts involving lifestyle management programs need a way to measure the support provided by the program and those conducting it, especially when the program is not influencing family support, a variable often measured during health behavior research. In this respect, more information is needed concerning the impact of support from health interventions apart from family support.

## Figures and Tables

**Table 1:** Demographic characteristics of the sample (*n* = 368).

	Mean	SD
**Age (18–79 years**)	33.9	14
	*n*	**%**
**State of Residence**		
Mississippi	238	64.7
Louisiana	130	35.3
**Gender**		
Female	276	75.0
Male	92	25.0
**Race/Ethnicity**		
White	160	43.5
Black/African American	113	30.7
American Indian/Alaskan Native	66	17.9
Mixed Race	19	5.2
Latino/Hispanic	5	1.4
Asian	4	1.1
Native Hawaiian/Other Pacific Islander	1	0.3
**Education Level**		
Less than a high school degree	14	3.8
A high school degree	48	13.0
Some college, but not a college degree	94	25.5
A 2 year or vocational degree	59	16.0
A 4 year college degree or higher	153	41.6
**Household Income**		
0–39,999	122	33.2
40,000–79,999	100	27.2
80,000–119,999	81	22.0
120,000 or greater	65	17.7
**Marital Status**		
Single	171	46.5
Married/Cohabitating	182	49.4
Divorced/Separated	15	4.1

**Table 2:** Reliability and descriptive data for information, emotional and encouragement social support to eat better and move more.

Social Support to Eat Better and Move More, α = 0.96 for all 10 Items	Mean	SD	Likert Item Response Distribution (%)						
	5.53	1.31	Strongly Disagree	Disagree	Somewhat Disagree	Neither Agree nor Disagree	Somewhat Agree	Agree	Strongly Agree
Informational Support to Eat Better and Move More	5.33	1.63							
When I need information to help me eat better or move more, …									
1. I have a friend who I can turn to for information.	5.21	1.71	5.2	6.5	3.3	10.1	23.9	24.5	26.6
2. I have a friend who will help me get the information I need.	5.31	1.68	5.4	4.9	3.5	8.2	22.3	28.8	26.9
3. I have a friend who will give me ideas/things to try.	5.46	1.65	5.4	4.3	2.4	6.3	18.5	34.0	29.1
Emotional Support to Eat Better and Move More	5.46	1.43							
When I am trying to eat better or be more active, …									
1. I have a friend who cares about how I am doing with my goals.	5.50	1.49	2.4	4.9	2.7	8.4	20.7	33.4	27.4
2. I have a friend who I can connect with who is trying to do the same thing as me.	5.46	1.51	3.0	5.2	1.9	9.5	19.3	35.9	25.3
3. I have a friend who helps me problem solve.	5.36	1.54	2.7	5.7	3.5	10.9	19.0	34.8	23.4
4. I have a friend who encourages me to meet my goal(s).	5.53	1.49	2.4	4.6	3.5	8.7	15.5	38.0	27.2
Encouragement to Eat Better and Move More	5.85	1.26							
When I meet my goals to eat better or be more active, …									
1. I have a friend who I can share my success with.	5.85	1.27	1.4	2.7	1.4	6.3	14.4	40.2	33.7
2. I have a friend who will celebrate my achievements with me.	5.80	1.32	1.1	3.5	2.4	6.5	13.3	39.9	33.2
3. I have a friend who will be proud of me.	5.90	1.29	1.4	3.3	0.8	5.4	13.6	38.6	37.0

**Table 3:** Simple correlations within and between social support subdomain items (1) to (10) followed by varimax-rotated principal components analysis loadings (a) and (b).

Cronbach’s α All 10 Items = 0.96	(1)	(2)	(3)	(4)	(5)	(6)	(7)	(8)	(9)	(10)	a	b
Informational Support (α = 0.97)												
(1) Informational Support—When I need information to help me eat better or move more, I have a friend who I can turn to for information.	**1**	0.926 **	0.893 **	0.680 **	0.721 **	0.717 **	0.720 **	0.617 **	0.626 **	0.595 **	**0.887**	0.318
(2) Informational Support—When I need information to help me eat better or move more, I have a friend who will help me get the information I need.		**1**	0.927 **	0.688 **	0.732 **	0.730 **	0.711 **	0.595 **	0.619 **	0.601 **	**0.907**	0.302
(3) Informational Support—When I need information to help me eat better or move more, I have a friend who will give me ideas/things to try.			**1**	0.681 **	0.711 **	0.710 **	0.713 **	0.605 **	0.610 **	0.604 **	**0.889**	0.309
Emotional Support (α = 0.96)												
(4) Emotional Support—When I am trying to eat better or be more active, I have a friend that cares about how I am doing with my goals.				**1**	0.857 **	0.836 **	0.887 **	0.695 **	0.728 **	0.691 **	**0.618**	**0.639**
(5) Emotional Support—When I am trying to eat better or be more active, I have a friend that I can connect with that is trying to do the same thing as me.					**1**	0.877 **	0.873 **	0.701 **	0.731 **	0.688 **	**0.668**	**0.612**
(6) Emotional Support—When I am trying to eat better or be more active, I have a friend who helps me problem solve.						**1**	0.857 **	0.655 **	0.689 **	0.655 **	**0.692**	**0.560**
(7) Emotional Support—When I am trying to eat better or be more active, I have a friend who encourages me to meet my goal(s).							**1**	0.731 **	0.771 **	0.737 **	**0.630**	**0.669**
Encouragement (α = 0.97)												
(8) Encouragement—When I meet my goals to eat better or be more active, I have a friend that I can share my success with.								**1**	0.921 **	0.905 **	0.318	**0.889**
(9) Encouragement—When I meet my goals to eat better or be more active, I have a friend who will celebrate my achievements with me.									**1**	0.936 **	0.338	**0.903**
(10) Encouragement—When I meet my goals to eat better or be more active, I have a friend who will be proud of me.										**1**	0.307	**0.898**

** *p* ≤ 0.01. Bolded correlations are correlations within subdomains.

**Table 4:** Convergent and predictive validity of Social Support to Eat Better and Move More.

	Value Labels	Social and Emotional Support	Total Added Sugars (tsp/day)	Fruits and Vegetables (cup/day)	Physical Activity (days/week)	Physical Health Score
Social Support (All 10 Items)	r	0.36	−0.16	0.13	0.15	0.22
	*p*	<0.01	<0.01	0.01	0.01	<0.01
Informational Support	r	0.31	−0.13	0.15	0.14	0.20
	*p*	<0.01	0.01	<0.01	0.01	<0.01
Emotional Support	r	0.35	−0.16	0.11	0.15	0.22
	*p*	<0.01	<0.01	0.03	<0.01	<0.01
Encouragement Support	r	0.36	−0.15	0.09	0.10	0.16
	*p*	<0.01	<0.01	0.08	0.05	<0.01

## Data Availability

The data presented in this study are available on request from the corresponding author. The data are not publicly available due to privacy issues.
